# Spatioselective functionalization of gold nanopillar arrays†

**DOI:** 10.1039/c9na00149b

**Published:** 2019-03-19

**Authors:** Claire Chattaway, Delphine Magnin, Etienne Ferain, Sophie Demoustier-Champagne, Karine Glinel

**Affiliations:** Institute of Condensed Matter & Nanosciences (Bio & Soft Matter), Université catholique de Louvain Croix du Sud 1, box L7.04.02 B-1348 Louvain-la-Neuve Belgium karine.glinel@uclouvain.be sophie.demoustier@uclouvain.be; it4ip S.A. Avenue Jean-Etienne Lenoir 1 B-1348 Louvain-la-Neuve Belgium

## Abstract

A process combining electrochemical nanofabrication by hard templating with the use of a masking strategy and surface functionalization methods, is developed to produce arrays of gold nanopillars of spatially-controlled surface chemistry. Therefore, a gold nanopillar array is first fabricated by performing metal electrochemical deposition into a track-etched membrane supported on a gold substrate. After dissolution of the membrane, a protective polymer layer is deposited on the array and partially etched to specifically reveal the top of the nanopillars. Then, a polythiolactone-based copolymer is grafted on the upper part of the nanopillars. Afterwards, the sacrificial polymer layer is dissolved to reveal the non-functionalized surface corresponding to the lower part of the gold nanopillars and the background surface. This surface is subsequently modified by a self-assembled monolayer (SAM) of alkylthiol molecules which leads to nanostructured surfaces with spatio-selective surface chemistry. The grafting of gold nanoparticles and of a bioadhesive peptide on the top and on the background of the nanopillar array, respectively, is performed to prove the versatility of the approach to produce bifunctionalized nanopillar arrays for biological, biosensing or (bio)catalysis applications.

## Abstract 2

Fabrication of gold nanopillar arrays with spatioselective surface functionalization.

## Graphical abstract

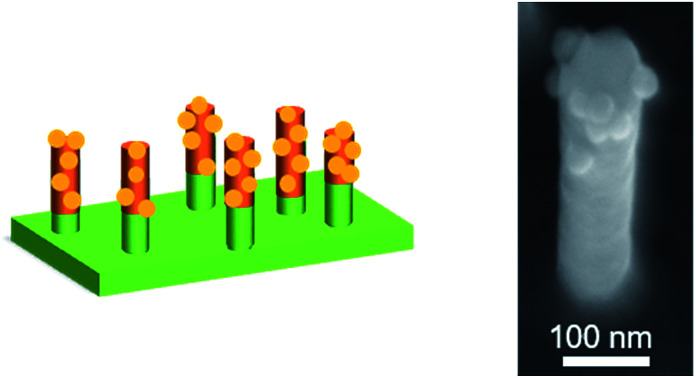

## 1. Introduction

Over the past two decades, there has been a great interest and active development in nanostructure-based devices because of the exceptional chemical and physical properties exhibited by nanoscale structures, leading to numerous and various potential applications.^1,2^ In particular, vertical semiconductor and metallic nanowire (NW) arrays, in which nanostructures are oriented perpendicular to the substrate, have attracted increasing research attention due to their unique structural features, such as high surface area, and excellent optical, electrochemical and electronic properties. Many applications of these nanodevices in the energy, catalysis, electronics, and especially in the biomedical and biotechnological fields, are developed.^3–6^ Their most notable bio-applications are biosensors, biochips for diagnostics, development of antibacterial surfaces, surface nano-structuration of medical implants, and drug delivery inside cells.^4,7–10^ These NW arrays also provide a nice opportunity for fundamental studies on the interfacial interactions between synthetic nanomaterials and living cells and tissues.^11^

Arrays of NWs with control over the dimensions, aspect ratio, density and location of the individual NW elements on the substrate can be generated by different bottom-up and top-down nanofabrication techniques.^12,13^ Among them, the templating strategy using either anodic aluminium oxide or polycarbonate track-etched templates is particularly attractive as it allows the fabrication of NW arrays or free individual nanowires with diameter, height and density finely and independently tunable over a broad range.^12,14^ Moreover, a large variety of single-component but also of multi-component nanowires, including segments of different chemical nature (polymer, metal or semiconductor) along the length of the nanowire can be easily synthesized by this templating technique.^15–17^

Besides the control of their physical dimension characteristics, surface (bio)-functionalization of the nanowires with one or several appropriate ligands or biomolecules are often required to tune these nanostructures towards a specific application. Up to now, surface modification was mostly applied onto the entire surface of the nanostructures.^18,19^ Though, spatio-selective functionalization of NW arrays with several active moieties offers a unique ability to combine a number of functionalities and/or properties while avoiding molecular interference due to randomly distributed functional groups that could lead to malfunction of the system. Chemical selective patterning of NW-based systems through NW site-specific functionalization with the respective ligands can be used to tailor surface properties, such as hydrophilicity, bio-adhesiveness or catalytic properties. Very recently, Huskens *et al.* reported on an original method for the spatioselective functionalization of silicon nanowires.^20^ The process combined metal-assisted chemical etching to fabricate silicon nanowires and hydrosilylation to form molecular monolayers. Click chemistry was then used for secondary modification of the monolayer with azide-functionalized nanoparticles. Regarding metallic NWs, the most frequently and successful reported spatioselective functionalization strategies rely on individual multi-component nanowires that are composed of different material segments.^21,23^ A variety of metal segments, such as Au, Ag, Cu, Co, Pd, Pt, Ni, have been incorporated into nanowires with either bimetallic or ternary configurations and each segment was derivatized with metal-specific chemistries.^22^ For instance, gold wires are most often functionalized with thiols, while nickel is most often functionalized with carboxylic acids, which bind to the native oxide layer on the metal. Several groups have successfully achieved various selective functionalization of multi-component nanowires.^15,16,21^ However, nanowires composed of a single metallic material, with a unique chemical affinity, cannot benefit from these selective surface functionalization procedures.

In the present work, we aimed for the spatio-selective functionalization of single-component metallic nanowire arrays. More specifically, a process combining electrochemical hard templating nanofabrication with surface functionalization methods and the use of a masking sacrificial polymer layer was designed and optimized for elaborating bifunctionalized gold nanopillar arrays. First, a protective layer was deposited by spin-coating on pre-fabricated Au nanopillar surfaces. Second, after a partial etching of the sacrificial polymer layer to reveal the upper surface of the nanopillars, a polythiolactone-based copolymer was grafted on the top of the nanopillars, according to a procedure that we recently reported.^24,25^ The further dissolution of the sacrificial polymer layer in water exposes the non-functionalized lower part of the gold nanopillars and the background surface, which were then differently modified through the formation of a self-assembled monolayer (SAM) of alkylthiol molecules. To prove the successful spatio-selective chemical surface modification of the Au nanopillar surfaces, the presence of the thiolactone-based copolymer was revealed through coupling of free thiol moieties with maleimide gold nanoparticles.

## 2. Materials and methods

### Materials

A statistical copolymer based on *N*,*N*-dimethylacrylamide (DMA) and thiolactone acrylamide (TlaAm) was synthesized by RAFT polymerization according to a methodology previously described.^23^ This P(DMA-*co*-TlAm) copolymer had an apparent average molar mass of 32 000 g moL^−1^ with a dispersity (*Đ*) of 1.1, as measured by size exclusion chromatography (SEC) and contained 25% of TlaAm units as determined by elemental analysis. Polycarbonate (PC) pellets (Lexan 145 from General Electrics), chloroform (CHCl_3_) (Sigma-Aldrich, 99.5+%, anhydrous), sodium hydroxide (NaOH) (Chem-Lab, 99+%), methanol (VWR, ACS grade), Dowfax™, gold(iii) chloride trihydrate (HAuCl_4_) (Sigma-Aldrich, 99.995%), potassium phosphate dibasic (K_2_HPO_4_) (Acros Organics, 99%), potassium chloride (KCl) (Acros Organics, 99+%), dichloromethane (CH_2_Cl_2_) (VWR, HPLC grade), polyacrylic acid (PAA, *M*_W_ = 100 000 g moL^−1^) (Sigma-Aldrich, 35% wt in water), ethanolamine (Sigma-Aldrich, 99%), ethanol (VWR, HPLC grade), 1-dodecanethiol (DDT) (Sigma-Aldrich, 98+%), 11-mercaptoundecanoic acid (MUA) (Sigma-Aldrich, 98%), 30 nm maleimide gold nanoparticles (NPs) conjugation kit (cytodiagnostics), FITC-labeled H-RGDK(FITC)-OH peptide (RGD-FITC, *M*_W_ = 863.91 g moL^−1^) (GeneCust, 99+%), sodium chloride (NaCl), (Sigma-Aldrich, 99+%), *N*-hydroxysulfosuccinimide sodium salt (sulfo-NHS) (Sigma-Aldrich, 98+%), *N*-(3-dimethylaminopropyl)-*N*′-ethylcarbodiimide hydrochloride (EDC) (Sigma-Aldrich, 98+%), 2-(*N*-morpholino)ethanesulfonic acid (MES) (Sigma-Aldrich, 99.5+%), sodium phosphate monobasic (NaH_2_PO_4_) (Acros Organics, 98+%), sodium phosphate dibasic (Na_2_HPO_4_) (Sigma-Aldrich, 99+%), hydrogen peroxide (Sigma-Aldrich, 30%), sulfuric acid (Sigma-Aldrich) were used as received.

Milli-Q water (resistivity 18.2 MΩ cm) was obtained from a Merck Millipore system (Milli-Q® Reference). Gold surfaces were fabricated in a clean room environment (Winfab platform, Université catholique de Louvain) by evaporating a 100 nm-thick layer of gold on silicon wafers (100) precoated with a 5 nm-thick layer of chromium. Single-side-polished (100) silicon wafers were purchased from TOPSIL. All glassware used for surface modification was cleaned using piranha solution (1 : 2 v/v mixture of hydrogen peroxide (30%) and sulfuric acid; *caution: piranha solution is extremely reactive and should be handled carefully*).

### Fabrication of gold nanopillar surfaces

Gold surfaces decorated with nanopillars were prepared by performing gold electrodeposition into the nanopores of a track-etched membrane deposited on a gold surface and used as a template.^26^ Briefly, in a first step, 1 × 1 cm^2^ silicon wafers coated with a 5 nm-thick chromium layer then a 100 nm-thick gold layer were cleaned with a piranha solution for 20 min, rinsed with Milli-Q water then ethanol and dried with a stream of N_2_. A 1 μm-thick polycarbonate film was deposited on these gold surfaces by spin-coating (4500 rpm, WS-400B-6NPP/Lite spin-coater, Laurell 8 Technologies Corporation) using a 95 mg mL^−1^ PC chloroform solution. The obtained samples were subsequently annealed at 190 °C for 6 h and beamed by heavy ions (Ar^9+^ at 220 MeV) at the Cyclotron Ressource Centre (Louvain-la-Neuve, Belgium). The surface of the samples was then UV-sensitized (wavelength 365 nm, 18 W) for 4 h at a power of 2 mW cm^−2^. Chemical etching was subsequently performed to open the latent ion tracks, by immersing the sensitized samples for 10 min in a 0.5 M NaOH methanol : water (50 : 50 v/v) solution, containing 1 : 1000 v/v of an anionic surfactant (Dowfax™) and heated at 52 °C. After a 2 min-rinsing in a methanol : water (50 : 50 v/v) solution, the surfaces were carefully dried with a stream of N_2_. The pore diameter and pore density obtained according to this protocol are typically 100 nm and 10^8^ cm^−2^, respectively.

Gold metal was then electrodeposited within the pores of the so-obtained track-etched supported membranes. For this, cyclic voltammetry was performed with a Chi660 instrument (ChInstruments) in presence of an aqueous solution containing 6 mM HAuCl_4_, 30 mM KCl and 30 mM K_2_HPO_4_. The reaction was performed at room temperature in a one-compartment Teflon® cell using a platinum counter electrode and an Ag/AgCl reference electrode. The gold surface covered with the track-etched PC membrane was used as the working electrode. The potential of this working electrode was linearly and reversibly ramped from 0 to 0.7 V at a scanning rate of 0.2 mV s^−1^. The number of scans was varied from 10 to 18 in order to tune the thickness of the gold layer deposited into the pores and consequently, the height of the resulting nanopillars.

The polycarbonate membrane was then dissolved by immersing the samples in CH_2_Cl_2_ then 0.5 M NaOH for 1 h. The nanopillar surfaces obtained were finally placed in a UV/ozone chamber (UVO-Cleaner, Jelight) for 20 min.

### Fabrication of bi-functionalized nanopillar surfaces

#### Step 1: Functionalization of the top of the gold nanopillars

A 0.05 mg mL^−1^ solution of PAA (pH 7) filtered through a 0.2 μm membrane (Acrodisc® syringe filter) was spin-coated at 1750 rpm on the gold nanopillar surfaces in order to cover the entire height of the nanopillars. The thickness of the resulting PAA layer deposited in the same conditions on a flat silicon wafer was 260 nm, as measured by ellipsometry. Then the top of the nanopillars was revealed by performing an air plasma treatment at 100 W for a given time. A PAA layer deposited on a silicon wafer and etched in the same conditions was measured by ellipsometry before and after the etching treatment, to estimate the decrease of the thickness of the polymer layer removed under treatment.

Then 5 mL of a P(DMA-*co*-TlAm) copolymer solution (0.2 mg mL^−1^) prepared in CHCl_3_ was placed in a glass vial. Ethanolamine was then added to get 2 eq. of TlaAm units for ethanolamine in the mixture.^23^ This mixture was stirred for 30 s and a freshly plasma-treated nanopillar surface was rapidly immersed in the solution. After a contact time of 2 h, the surface was thoroughly rinsed with CHCl_3_, ethanol then Milli-Q water and dried with a stream of N_2_. The PAA layer remaining on the surface was dissolved by dipping the sample four times in an aqueous solution (pH 7) for 5 min and one more time for 10 min. Then the surface was thoroughly rinsed with Milli-Q water and dried.

#### Step 2: Functionalization of the background of gold nanopillar surfaces

After dissolution of the PAA mask, the nanopillar surface with the top of the nanopillars functionalized with P(DMA-*co*-TlAm) was immersed overnight in an ethanol solution containing 20 mM of DDT or MUA, in order to produce a SAM on the background of the surface. Surface was then thoroughly rinsed with ethanol and dried with a stream of N_2_.

### Selective immobilization of gold nanoparticles bearing maleimide groups on the top of the P(DMA-*co*-TlAm)-grafted nanopillars

Gold NPs bearing maleimide groups on their surface were dispersed in a mixture of 55 : 45 v/v (reaction buffer : resuspension buffer) provided by the supplier, at a concentration of 44 mg mL^−1^. 30 μL of this solution were deposited on a nanopillar surface having nanopillars grafted with P(DMA-*co*-TlAm) in order to immobilize the gold NPs on the free thiol groups of the copolymer through the maleimide–thiol click reaction. The click reaction was carried out for 1 h then the surface was gently but thoroughly rinsed with Milli-Q water, ethanol and dried with a stream of N_2_.

### Grafting of RGD-FITC peptide on a SAM of MUA

Gold surfaces modified with a SAM of MUA were immersed for 15 min in a freshly prepared 0.1 M MES buffer (0.5 M NaCl, pH 6) containing 2 mM EDC and 5 mM sulfo-NHS to activate the carboxylic groups of the MUA layer. After rinsing with MES buffer, the surfaces were immersed in a 0.1 M phosphate buffer (pH 8) containing RGD-FITC peptide (0.6 mM). After a reaction time of 2 h, the surfaces were rinsed with Milli-Q water, sonicated in an ultrasound bath for 1 min then rinsed with ethanol and dried under a stream of N_2_.

### Characterization techniques

#### Scanning electron microscopy (SEM)

A FEG-SEM 7600 F from Jeol was used (with a working distance of 8 mm, 15 kV) to observe the gold nanopillar surfaces.

#### Ellipsometry

A single wavelength (658 nm) and variable angle EP3 ellipsometer from Accurion was used to measure the thickness of PAA layers spin-coated on silicon wafers, before and after plasma treatment, in order to estimate the decrease of the layer thickness after etching. The data were analyzed with EP4 Model software by using a 3 layer model comprising a flat silicon substrate with a refractive index of 3.840-j0.016, a native silicon oxide layer with a refractive index of 1.456 and a PAA layer with a refractive index of 1.50.

#### Atomic force microscopy (AFM)

AFM images of the nanopillar surfaces were recorded in Tapping™ mode (in light tapping regime) with a Bruker Nanoscope V device equipped with a high aspect ratio tip (ART5-NCHR-10 from Nanosensors). They were then analyzed using NanoScope Analysis 1.8 software (Bruker). The recorded images were systematically flattened by a 1^st^ order polynomial function.

#### Epifluorescence microscopy

The gold surfaces grafted with the fluorescent RGD-FITC peptide were observed with an optical inverted epifluorescence IX71 microscope (Olympus, Japan) equipped with a CCD camera and a fluorescence illuminator and various mirror units. Images were recorded at 40 × magnification and processed using the Cell Sens Dimension 1.7 software (Olympus, Japan).

## 3. Results and discussion

**Scheme 1:**
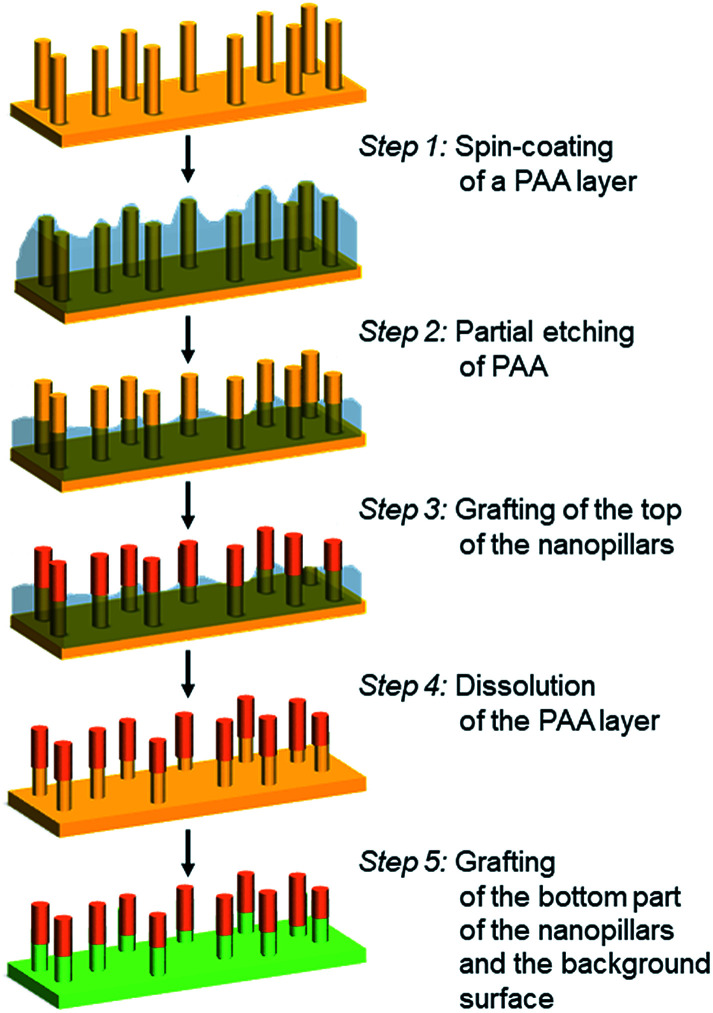
Description of the process used to prepare bifunctionalized gold nanopillar surfaces: spin-coating of a PAA layer on nanopillar surface (step 1); partial etching of the PAA layer to reveal the top of the nanopillars (step 2); grafting of a copolymer layer on the top of the nanopillars (step 3); dissolution of the remaining PAA layer (step 4); grafting of a SAM on the bottom part of the nanopillars and on the background surface (step 5).

The elaboration of bifunctionalized gold nanopillar surfaces was developed according to a 5-step process described in Scheme 1. First the nanopillar surface is covered by a PAA layer by spin-coating (step 1). Then the top part of the nanopillars is revealed by a partial etching of the PAA layer (step 2). The top of the nanopillars is subsequently chemically grafted (step 3) and the remaining PAA layer is dissolved in water (step 4) in order to allow the chemical modification of bottom part of the nanopillars and the background surface (step 5).

### Deposition of a PAA layer on nanopillar surfaces

**Fig. 1:**
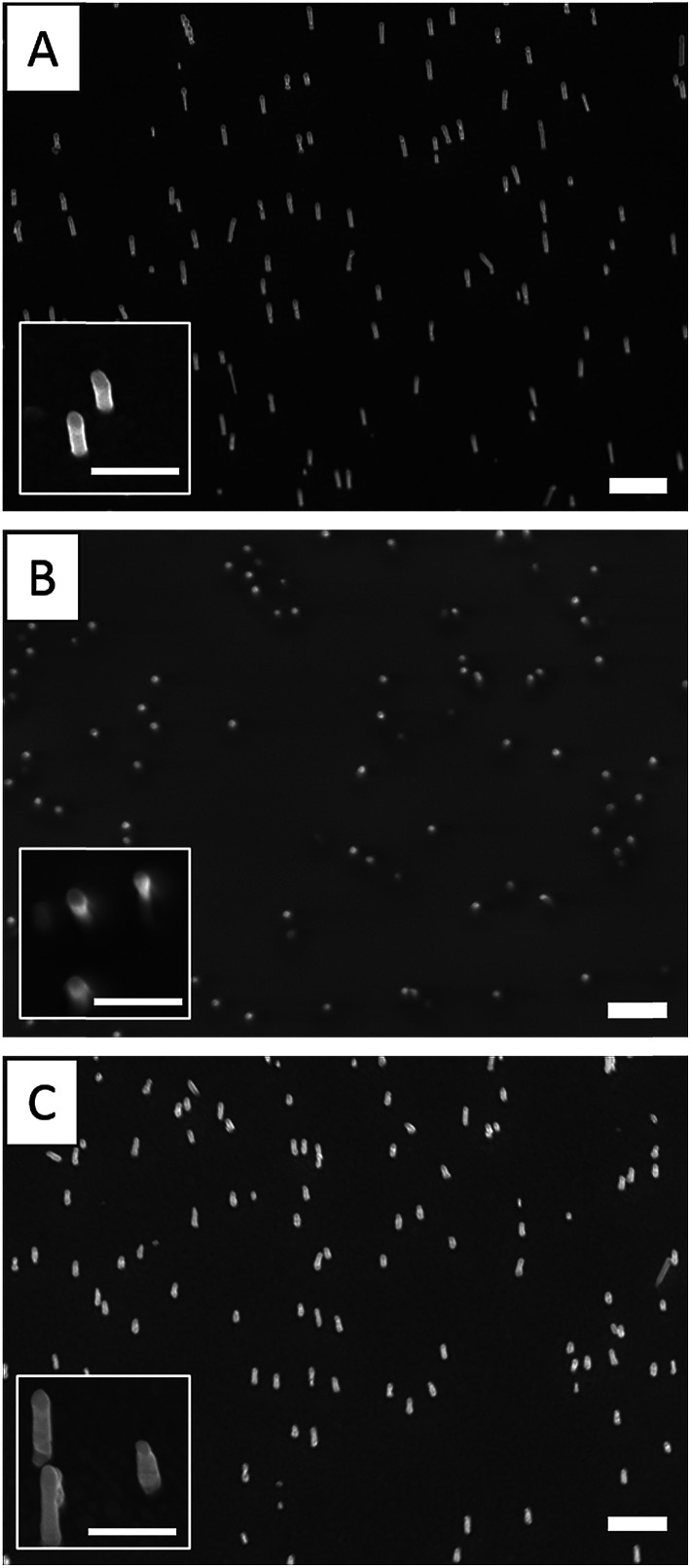
SEM images of a gold surface decorated with 500 nm-height nanopillars before the deposition of a PAA layer (A), after deposition of spin-coated PAA layer (B) and after the dissolution of the PAA layer in aqueous solution (C). Scale bars represent 1 μm in the main images and 500 nm in the insets.

By varying the number of voltammetry cycles performed during the gold electrodeposition into the nanopores of PC supported membranes, nanopillar surfaces with a nanopillar height varying from 365 ± 68 nm to 950 ± 160 nm were obtained, as measured by SEM (results not shown). In the second step, these surfaces were submitted to spin-coating at different speeds in order to explore the possibility to deposit a PAA layer on these surfaces by this technique. It was shown that the higher nanopillars tended to bend or to lie onto the surface due to their low mechanical resistance towards the centrifugal force applied during spin-coating, even when a slow speed was used. Therefore only the surfaces decorated with nanopillars with a height lower than 800 nm were used in the next steps of the functionalization process. Using a spin-coating speed of 1750 rpm allowed to keep upright 500 nm-height nanopillars as observed by SEM (Fig. 1). A PAA film, with an equivalent thickness of 260 nm on flat silicon substrate (as measured by ellipsometry), was successfully deposited on such a surface as shown in Fig. 1B. Moreover the PAA layer could be easily dissolved in aqueous solution without any damage observed on the nanopillars as shown by SEM (Fig. 1C).

**Fig. 2:**
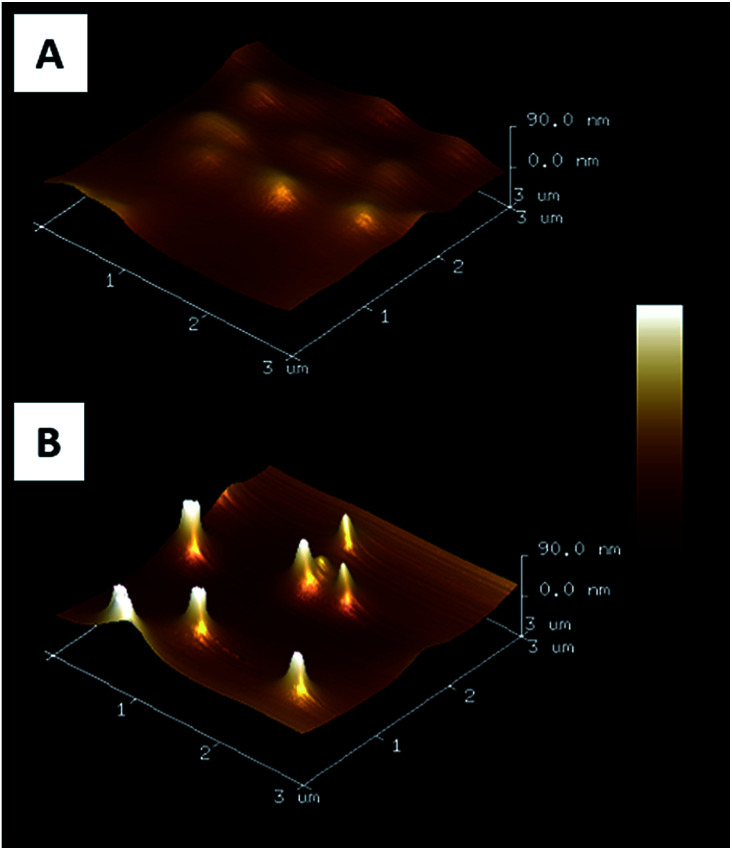
AFM 3D images (3 × 3 μm^2^) of gold nanopillars covered by a spin coated PAA film. Nanopillar heights are 365 ± 68 nm (A) and 477 ± 105 nm (B).

AFM images of nanopillar surfaces covered by a spin-coated PAA layer were recorded to get a better view of the morphology of these surfaces. These measurements were done on surfaces decorated with nanopillars with a height of 477 ± 105 and 365 ± 68 nm (Fig. 2). The surface decorated with the smaller nanopillars (Fig. 2A), showed a relatively smooth morphology and the presence of the nanopillars under the PAA layer resulted in the presence of small bumps on the surface. In contrast, the surface decorated with the higher nanopillars (Fig. 2B), displayed clear protrusions covered by a thin polymer film. Image analysis performed on these surfaces revealed that the height of these protrusions ranged from 50 to 300 nm (Fig. S1A†). So, even if the theoretical thickness of the PAA layer (measured on flat surface) is lower than the height of the nanopillars, the PAA layer is deposited on the entire surface with a higher thickness on the background compared to the top of the nanopillars.

To reveal the top of the nanopillars in order to modify them, the PAA covered surfaces underwent an air plasma treatment to partially remove the PAA layer. For this, a calibration was performed on 260 nm-thick PAA layers spin-coated on silicon wafers then exposed to an air plasma for various times. The thickness of the resulting layers was systematically measured by ellipsometry to estimate the efficiency of the etching (Fig. S2†). It was shown that a continuous plasma treatment for 150 s allowed to remove a PAA thickness of 20 nm. However large damages and cracks were observed by AFM on such treated films (results not shown). For this reason, two subsequent treatments of 120 s were performed which resulted in a decrease of film thickness of 17 nm, without damaging the film. These conditions were thus used to treat surfaces decorated with nanopillars having height of about 500 nm and coated with a PAA layer. The AFM images recorded before and after the plasma treatment revealed that the distribution of the size of the protrusions was displaced to the larger sizes after the treatment which confirmed the partial etching of the PAA layer (Fig. S1†) allowing to reveal the top of the nanopillars and their subsequent grafting.

### Grafting of a copolymer layer on the top of the nanopillars

**Scheme 2:**
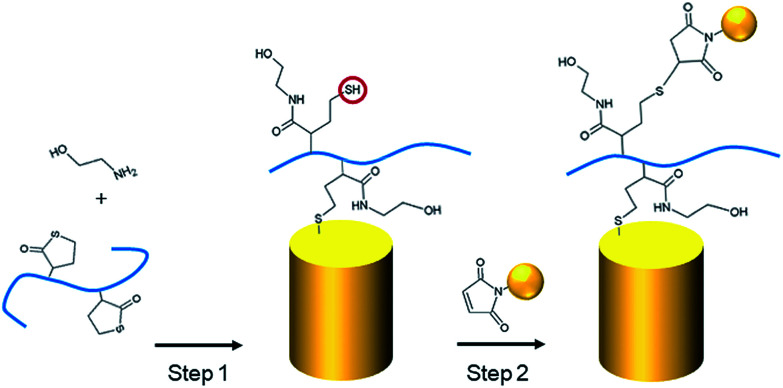
Grafting of the P(DMA-*co*-TlAm) copolymer on gold nanopillars in presence of ethanolamine (step 1) and immobilization of gold NPs bearing maleimide groups on their surface, on the thiol groups of the resulting layer (step 2).

As described in our previous studies,^24,25^ a P(DMA-*co*-TlAm) copolymer layer can be efficiently grafted on a gold surface in presence of an amine derivative such as ethanolamine (Scheme 2, step 1). The copolymer grafting is carried out through the pendent thiol groups which are released from the thiolactone rings under the nucleophilic addition of the amine derivative. Moreover, it was shown that only a fraction of the copolymer thiol groups is involved in the formation of S–Au links with the surface, while the other fraction remains free to perform post-modification of the layer.^24^

**Fig. 3:**
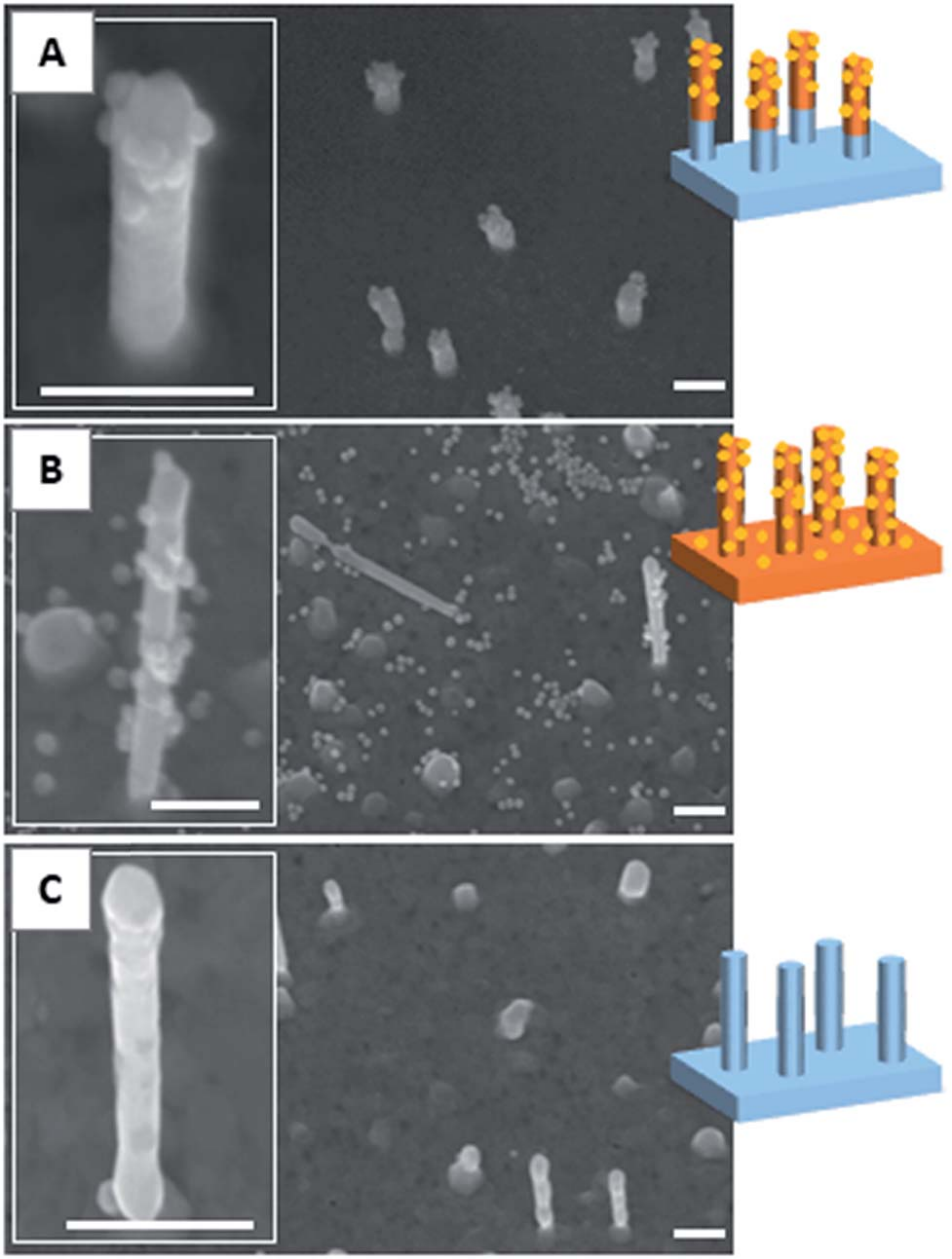
SEM images showing gold nanopillar surfaces after different modifications: (A) bifunctionalized surface with the top of the nanopillars grafted with a thiolactone copolymer layer post-modified with maleimide gold NPs and the background modified by a DDT SAM; (B) surface entirely grafted with a thiolactone copolymer layer then post-modified with maleimide gold NPs; (C) surface entirely functionalized with a SAM of DDT then exposed to maleimide gold NPs to evidence the non-adsorption of these NPs on such a layer. The height of the nanopillars is 660 ± 110, 850 ± 180 and 940 ± 200 nm in (A, B and C) images, respectively. Scale bars in the main images and in the insets represent 200 nm.

The grafting of the copolymer layer in the presence of ethanolamine was first tested on a bare gold nanopillar surface. To evidence the presence of the copolymer layer onto the surface, gold NPs bearing maleimide groups on their surface were reacted with the free thiol groups of the copolymer layer according to the Michael addition described in Scheme 2 (step 1).^27^ As shown in Fig. 3B, the NPs were observed on both nanopillars and background of the surface, testifying for their successful grafting on the free thiol groups of the copolymer layer. In a second step, a similar experiment was performed with the copolymer layer present only on the top of the gold nanopillars. For this, a nanopillar surface spin-coated with a PAA layer then exposed to air plasma, was immersed in a copolymer solution in presence of ethanolamine in order to functionalize the top of the nanopillars. Then the remaining PAA was dissolved in aqueous solution and the resulting surface was immersed in the gold maleimide NPs suspension in order to achieve the selective immobilization of these NPs on the free thiol groups of the copolymer layer. SEM observations performed on this sample revealed that NPs were clearly immobilized on the top of the nanopillars but a small amount was also adsorbed on the bare gold background (results not shown). Therefore, to avoid such an artifact and to evidence the specific grafting of the NPs on the top of the nanopillars, the passivation of the gold background was achieved before the addition of the NPs. For this, a gold surface with the top of the nanopillars grafted with the copolymer layer was exposed to a DDT solution in order to form a SAM on the bare gold background to passivate it. A control experiment consisting of adding NPs bearing maleimide groups on a nanopillar surface entirely covered by a SAM of DDT evidenced that this layer avoided any deposition of NPs (Fig. 3C) and thus could be successfully used to passivate the surface background. Therefore, the surface with the top of the nanopillars modified with the copolymer and the background with DDT, was subsequently exposed to the NPs. As seen on the SEM images displayed in Fig. 3A, the NPs were observed only on the top of the nanopillars and not on the DDT background. This result proves the structure of the surface with the top of the nanopillars grafted with the copolymer layer and the background with the DDT layer.

### Preparation of bifunctionalized nanopillar surfaces

**Fig. 4:**
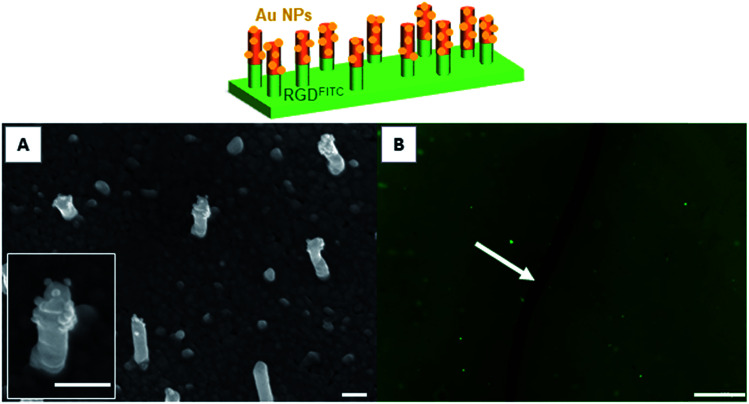
SEM (A) and epifluorescence microscopy images (B) of a bifunctional nanopillar surfaces bearing a copolymer layer grafted with gold maleimide NPs on the top of the nanopillars and a MUA layer grafted with a RGD-FITC peptide on the bottom part of the nanopillars and on the background surface. A scratch (indicated by a white arrow in (B)) was deliberately made on the modified surface to distinguish the fluorescent intensity resulting from the fluorescent layer and the background. Moreover the contrast of image (B) was increased by 15%. Scale bars represent 200 nm and 200 μm in SEM (A) and epifluorescence images (B), respectively.

To prepare nanopillar surfaces showing two different chemical functions located on the top of the nanopillars and on the background, respectively, we proceeded as described above while replacing the DDT by MUA in order to form a SAM background ended by carboxylic acid functions.^28^ After immobilization of the gold maleimide NPs on the copolymer layer located on the top of the nanopillars, the MUA background was grafted with a fluorescent RGD-FITC peptide through an EDC/sulfo-NHS activation. The obtained surface was observed by fluorescence microscopy which clearly revealed the presence of the fluorescent peptide (Fig. 4B) on the background surface. Moreover the SEM observation confirmed the presence of the gold NPs only on the top of the nanopillars (Fig. 4A). No decrease of the background fluorescence or detachment of gold NPs were observed after storing the surfaces for 24 h in buffer solution, testifying for the stability of the bifunctionalized surfaces. Control experiments were also performed by incubating flat gold surfaces entirely covered by a MUA layer with the RGD-FITC peptide, with and without EDC/sulfo-NHS activation. Interestingly, no fluorescence signal was detected on the non-activated surface while a clear fluorescent signal was observed for the activated surface (Fig. S3†). Altogether these experiments proved that the RGD-FITC peptide was not simply adsorbed but chemically grafted on the MUA background when bifunctionalized nanopillar surfaces were prepared. Moreover, to prove that the RGD-FITC peptide was not grafted on the top of the nanopillars, a gold nanopillar surface entirely covered with a thiolactone copolymer layer grafted with gold maleimide nanoparticles was exposed first to EDC/sulfo-NHS then to RGD-FITC, according to the activation sequence used to prepare the biofunctionalized surfaces. The epifluorescence microscopy measurements performed on this surface revealed the absence of fluorescence (Fig. S4†), proving that the RGD peptide cannot be grafted on the top of the nanopillars present of the bifunctionalized surfaces.

## 4. Conclusion

Over the last decades, a number of nanofabrication techniques such as e-beam lithography, scanning probe lithography, photolithography, focus ion beam lithography, and hard and soft templating was used in combination with surface grafting methods to produce nanostructured surfaces homogeneously covered with various functional layers. However, the development of nanostructured surfaces with a spatio-selective functionalization of the nanostructures remains scarce.

In this work, we successfully produced gold nanostructure arrays with three levels of functionalization: (1) nanostructure topography in the form of gold nanowires, (2) selective chemical modification of the top of the nanowires, (3) selective chemical modification of the bottom of the nanowires and substrate. The fabrication process used to prepare these surfaces is relatively simple, does not require a clean room environment or expensive equipment. Using polymer track-etched membranes as templates allows to produce large surfaces of nanostructures with dimensions (diameter and height) and density that can be independently and finely tuned by adapting the processing conditions. Moreover, the strategy can be generalized to the grafting of many different ligands on the nanostructures and might also allow the preparation of smart surfaces by performing a spatio-selective grafting of stimuli-responsive molecules on the arrays. Therefore, we expect that the spatio-selective surface functionalization approach we developed here will be extremely valuable for the study of confined surface mechanisms involved in catalysis, bioadhesion, plasmonics or energy transfer.

## Conflicts of interest

There are no conflicts of interest to declare.

## Supplementary Material

NA-001-C9NA00149B-s001

## References

[cit1] Tawfick S., De Volder M., Copic D., Park S. J., Oliver C. R., Polsen E. S., Roberts M. J., Hart A. J. (2012). Adv. Mater..

[cit2] Sannicolo T., Lagrange M., Cabos A., Celle C., Simonato J. P., Bellet D. (2016). Small.

[cit3] He B., Yang Y., Yuen M. F., Chen X. F., Lee C. S., Zhang W. J. (2013). Nano Today.

[cit4] Elnathan R., Kwiat M., Patolsky F., Voelcker N. H. (2014). Nano Today.

[cit5] Xie C., Hanson L., Cui Y., Cui B. (2011). Proc. Natl. Acad. Sci. U. S. A..

[cit6] Lis D., Caudano Y., Henry M., Demoustier-Champagne S., Ferain E., Cecchet F. (2013). Adv. Opt. Mater..

[cit7] Yu Q., Liu H., Chen H. (2014). J. Mater. Chem. B.

[cit8] He Y., Fan C., Lee S. T. (2010). Nano Today.

[cit9] Bucaro M. A., Vasquez Y., Hatton B. D., Aizenberg J. (2012). ACS Nano.

[cit10] Susarrey-Arce A., Sorzabal-Bellido I., Oknianska A., McBride F., Beckett A. J., Gardeniers J. G. E., Raval R., Tiggelaar R. M., Diaz Fernandez Y. A. (2016). J. Mater. Chem. B.

[cit11] Bonde S., Buch-Manson N., Rostgaard K. R., Andersen T. K., Berthing T., Martinez K. L. (2014). Nanotechnology.

[cit12] Nesbitt N. T., Naughton M. J. (2017). Ind. Eng. Chem. Res..

[cit13] Weisse J. M., Lee C. H., Kim D. P., Zheng X. (2012). Nano Lett..

[cit14] Cao G., Liu D. (2008). Adv. Colloid Interface Sci..

[cit15] Hurst S. J., Payne E. K., Qin L., Mirkin C. (2006). Angew. Chem., Int. Ed..

[cit16] Roy C. J., Chorine N., De Geest B., De Smedt S., Jonas A. M., Demoustier-Champagne S. (2012). Chem. Mater..

[cit17] Callegari V., Demoustier-Champagne S. (2011). Macromol. Rapid Commun..

[cit18] Conde J., Dias J. T., Grazu V., Moros M., Baptista P. V., de la Fuente J. M. (2014). Front. Chem..

[cit19] De Smet L. , Uliien D., Mescher M. and Sudhoelter E., in Nanowires – Implementations and Applications, ed. A. A. Hashim, IntechOpen, Rijeka, Croatia, 2011, vol. 13, pp. 267–288

[cit20] Verbeek J., Huskens J. (2018). ChemNanoMat.

[cit21] Wildt B., Mali P., Searson P. C. (2006). Langmuir.

[cit22] Pearce M. E., Melanko J. B., Salem A. K. (2007). Pharm. Res..

[cit23] Oh T., Ku J. C., Ozel T., Mirkin C. A. (2017). J. Am. Chem. Soc..

[cit24] Belbekhouche S., Reinicke S., Espeel P., Du Prez F. E., Dupont-Gillain C., Jonas A. M., Demoustier-Champagne S., Glinel K. (2014). ACS Appl. Mater. Interfaces.

[cit25] Chattaway C., Belbekhouche S., Du Prez F. E., Glinel K., Demoustier-Champagne S. (2018). Langmuir.

[cit26] Dauginet-De Pra L., Ferain E., Legras R., Demoustier-Champagne S. (2002). Nucl. Instrum. Methods Phys. Res., Sect. B.

[cit27] Shimada K., Mitamura K. (1994). J. Chromatogr. B: Biomed. Sci. Appl..

[cit28] Stettner J. , Frank P., Griesser T., Trimmel G., Schennach R., Resel R. and Winkler A., Interface Controlled Organic Thin Films, in Springer Proceedings in Physics, ed. K. Al-Shamery, G. Horowitz, H. Sitter and H. G. Rubahn, Springer, Berlin, Heidelberg, Germany, 2009, vol. 129, pp. 101–105

