# Supplementary material for: Spatioselective functionalization of gold nanopillar arrays
Source: Nanoscale Adv. 2019 Mar 19;1(6):2208–15. doi: 10.1039/c9na00149b (PMC9418540; doi:10.1039/c9na00149b)
Supplement: NA-001-C9NA00149B-s001 [file NA-001-C9NA00149B-s001.pdf]

Electronic Supplementary Material (ESI) for Nanoscale Advances.
This journal is © The Royal Society of Chemistry 2019

Spatioselective functionalization of gold nanopillar arrays

Claire Chattaway,[1] Delphine Magnin,[1] Etienne Ferain,[1,2] Sophie Demoustier-Champagne, [1,*] Karine Glinel[1,*]

[1] Institute of Condensed Matter & Nanosciences (Bio & Soft Matter), Université catholique de Louvain, Croix du Sud 1, box L7.04.02, B-1348 Louvain-la-Neuve, Belgium.

[2] it4ip S.A., avenue Jean-Etienne Lenoir 1, B-1348 Louvain-la-Neuve, Belgium.

Corresponding Authors
*Email: karine.glinel@uclouvain.be; sophie.demoustier@uclouvain.be

Supplementary Information

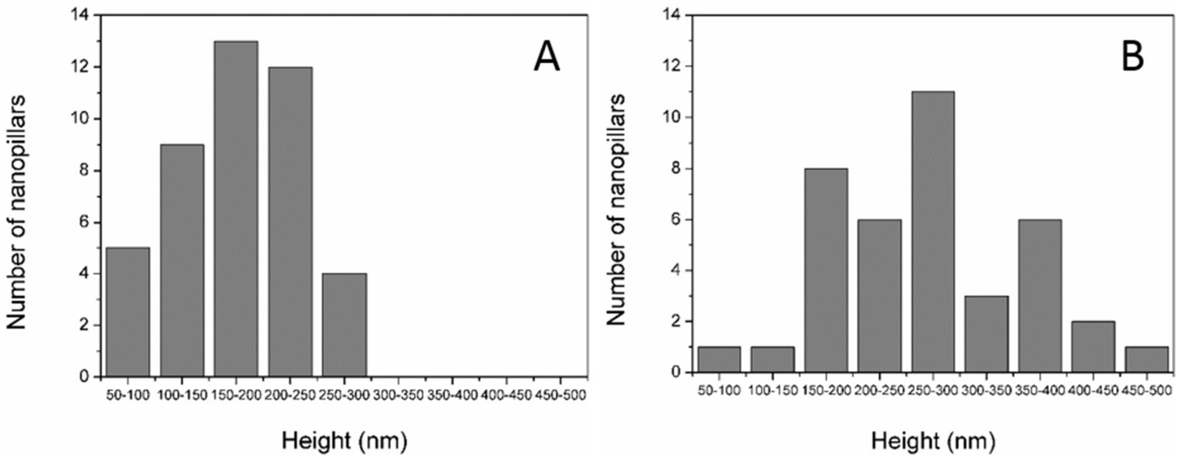

Figure S1. Histograms showing the height distribution of the protruding parts of nanopillars out of the PAA layer evaluated by AFM before (A) and after (B) a 2x120 sec air-plasma etching (B). The height of the gold nanopillars is 477 ± 105 nm.

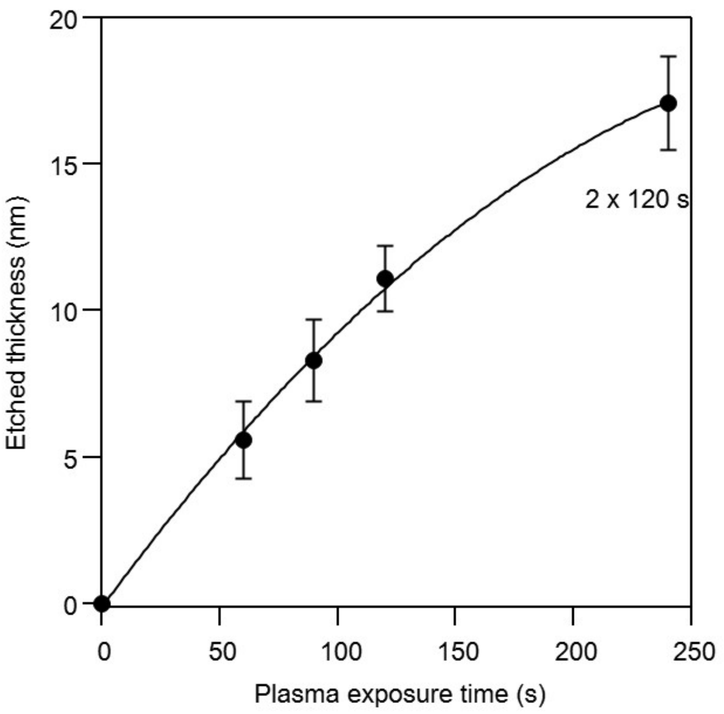

Figure S2. Variation of the PAA layer thickness etched as a function of exposure time to air plasma. The initial thickness of the PAA layer, before exposure, is 260 nm.

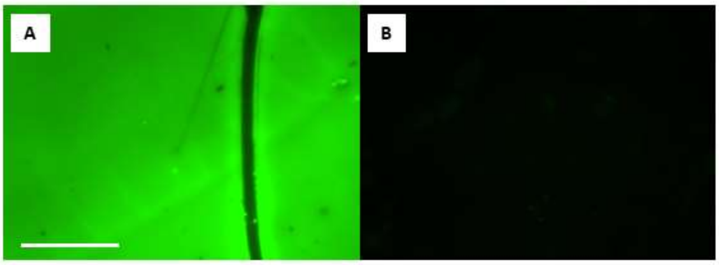

Figure S3. Epifluorescence microscopy images of flat gold surfaces covered with a MUA layer activated (B) or not (A) by EDC/sulfo-NHS then incubated with RGD-FITC peptide. The black line seen in image (B) is a deliberated scratch done to show the fluorescence intensity of the grafted layer. The scale bar represents 200 µm.

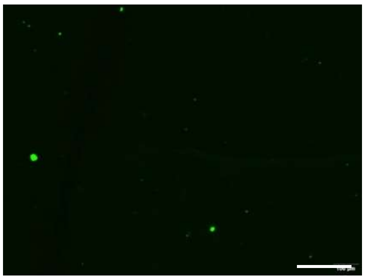

Figure S4. Epifluorescence microscopy image of a gold nanopillar surface entirely covered with a thiolactone copolymer layer grafted with maleimide gold nanoparticles, incubated with EDC/sulfo-NHS then RGD-FITC. The scale bar represents 200 µm.
